# Sensitive detection of type G botulinum neurotoxin through Endopep-MS peptide substrate optimization

**DOI:** 10.1007/s00216-019-01926-8

**Published:** 2019-06-06

**Authors:** Dongxia Wang, Jakub Baudys, Kaitlin Hoyt, John R. Barr, Suzanne R. Kalb

**Affiliations:** Division of Laboratory Sciences, Centers for Disease Control and Prevention, National Center for Environmental Health, 4770 Buford Hwy, N.E., Atlanta, GA 30341 USA

**Keywords:** Botulinum neurotoxin, Botulism, Mass spectrometry, Peptide substrate

## Abstract

*Clostridium botulinum* produces botulinum neurotoxins (BoNTs) that are one of the most poisonous substances. In order to respond to public health emergencies, there is a need to develop sensitive and specific methods for detecting botulinum toxin in various clinical matrices. Our laboratory has developed a mass spectrometry-based Endopep-MS assay that is able to rapidly detect and differentiate BoNT serotypes A–G by immunoaffinity capture of toxins and detection of unique cleavage products of peptide substrates. To improve the sensitivity of the Endopep-MS assay for the detection of BoNT serotype G, we report here the optimization of synthetic peptide substrates through systematic substitution, deletion, and incorporation of unnatural amino acids. Our data show that the resulting optimized peptides produced a significant improvement (two orders of magnitude) in assay sensitivity and allowed the detection of 0.01 mouseLD_50_ toxin present in buffer solution.

## Introduction

Botulinum neurotoxins (BoNTs) are highly toxic bacterial protein toxins produced by *Clostridium botulinum*, *C*. *butyricum*, *C*. *argentinese*, and *C*. *baratii*. Intoxication with these toxins can lead to an inhibition of neurotransmitter release at the neuromuscular junction and result in flaccid paralysis producing a life-threatening clinical condition called botulism [1, 2]. BoNTs constitute a potential biological weapon due to their extremely high toxicity and ease of manufacture [3]. On the other hand, BoNTs have also been used in therapy and cosmetics [4]. Good public health practice necessitates a simple, fast, and sensitive way to detect these toxins.

There are seven confirmed serotypes of BoNTs (A–G) based on their antigenic properties, and many serotypes contain several subtypes or variants. All of the BoNTs are synthesized as single-chain polypeptides. After proteolytic cleavage, active toxins are generated and consist of two separate chains tethered by a disulfide bond. The heavy chain of 100 kDa is involved in target binding and cell entry; the light chain of 50 kDa serves as a zinc-dependent endopeptidase and specifically cleaves one or two of three SNARE proteins involved in the vesicular fusion process. BoNT/A and BoNT/E cleave SNAP (synaptosomal-associated protein)-25, whereas BoNT/B, BoNT/D, BoNT/F, and BoNT/G target synaptobrevin-2(also known as VAMP-2) and BoNT/C cleaves both SNAP-25 and syntaxin [5–12].

Utilizing the hydrolysis activity of BoNT light chains, several functional assays have been developed for the detection of toxins in samples. A typical procedure includes the immunoaffinity separation and concentration of BoNTs in samples, followed by toxin hydrolysis of synthetic peptide substrates that mimic their native substrates. The cleavage products generated can then be measured by different detection platforms: fluorescence sensors, surface plasmon resonance, high-performance liquid chromatography (HPLC), mass spectrometry (MS), and other analytical techniques [13–18]. Our laboratory has developed a MS-based activity assay known as Endopep-MS which can detect and differentiate all seven serotypes of BoNTs present in various matrices [19–24]. Due to its high specificity and sensitivity, this method is being implemented in several national and international public health laboratories.

*C*. *botulinum*type G has been identified in soil samples, and BoNT/G was identified in post-mortem human samples, although the patients were not identified as suffering from botulism [25, 26]. Based on the similarity in amino acid sequence, protein receptor, and substrate recognition, BoNT/G is a close neighbor to serotype B. The two proteins share 57.9% amino acid homology, whereas the similarity of BoNT/G to other BoNT serotypes is significantly less, ranging from 32.5 to 38% [27]. Both toxins bind two homologous membrane-anchored proteins in synaptic vesicles, i.e., Syt-I and Syt-II, for their translocation into nerve cells [28–30]. The cleavage sites on the endogenous substrate, VAMP-2, are only a few amino acids apart as BoNT/B cleaves at Gln_76_-Phe_77_ and BoNT/G hydrolyzes at Ala_80_-Ala_81_ [9, 12]. In the Endopep-MS method, we previously used one peptide substrate derived from the sequence of VAMP-2_60–94_ that covered the two cleavage sites for the detection of both BoNT/B and BoNT/G toxins [19]. To improve the detection limits for BoNT/G, we analyze the effect of substrate peptide length and individual amino acid residues on the detection of the cleavage products of tested peptides and optimize the performance of peptide substrates. After residue substitution, deletion, and incorporation of unnatural amino acids, the optimal BoNT/G-specific substrate led to an increase in the sensitivity of the assay to detect active BoNT/G by two orders of magnitude.

## Materials and methods

All chemicals were obtained from Sigma–Aldrich (St. Louis, MO) unless otherwise stated. Fmoc-amino acid derivatives and peptide synthesis reagents were purchased from EMD Chemicals, Inc. (Gibbstown, NJ) or Protein Technologies (Tucson, AZ). Stable isotope-labeled Fmoc-amino acid derivatives were purchased from Cambridge Isotope Laboratories (Tewksbury, MA). The complex form of all botulinum neurotoxins was obtained from Metabiologics (Madison, WI). The specific activity of the BoNT/G complex was provided by the manufacturer to be about 1.7 × 10^4^ mouse LD_50_ Units/mg. Botulinum neurotoxin is highly toxic and appropriate safety measures are required. All BoNT neurotoxins were handled in a class II type A2 biosafety cabinet equipped with HEPA filters.

### Peptide synthesis

All peptides were prepared in house on a Liberty Blue microwave peptide synthesizer (CEM, Matthews, NC, USA) by a solid-phase peptide synthesis method using Fmoc chemistry. A mixture of 95% trifluoroacetic acid:2% water:2% anisole:1% ethanedithiol was used for side-chain deprotection and final cleavage from the resin. The synthetic peptides were purified by HPLC with a semi-preparative reverse phase C18 column using a water:acetonitrile:0.1% TFA (trifluoroacetic acid) gradient. The correct peptide structures were confirmed by matrix-assisted laser desorption/ionization time of flight (MALDI) mass spectrometry. All peptides were dissolved in deionized water as a 1 mM stock solution and were stored at − 70 °C until further use.

### Endopep-MS assay

Endopep-MS assay is a method developed in our laboratory to rapidly detect and differentiate all seven BoNT serotypes by affinity-concentrating toxins and detecting the unique serotype-specific cleavage products of peptide substrates. The peptides are optimized mimics of the sequence of the SNARE proteins that are the native substrates of BoNT. Endopep-MS analysis was performed as previously described [31]. Briefly, reactions were conducted at 42 °C for 1–4 h in a 20 μL reaction volume containing 0.1 mM peptide substrate, 10 μM ZnCl_2_, 1 mg/mL BSA, 10 mM dithiothreitol, and 200 mM HEPES buffer (pH 7.4). Different concentrations of BoNT/G were added directly to the reaction mixture as previously described [31]. No product peaks were detected from negative controls where no toxin was spiked in the sample solutions.

After the reaction, 2 μL of the supernatant was mixed with 20 μL of 5 mg/mL of α-cyano-4-hydroxy cinnamic acid in 50% acetonitrile/0.1% TFA/1 mM ammonium citrate; 2 μL of a 0.5 μM internal standard peptide was added to the solution. The internal standard is a stable isotope-labeled peptide resembling the sequence of the C-terminal cleavage product of the peptide substrate tested, with the sequence of AKL(+7) KRRYWWAKL and the molecular weight of 1625.0 Da. The formation of cleavage products was measured as a ratio of the cleavage product versus the internal standard. Data are an average of three replicate experiments.

### MS detection

Each sample was spotted in triplicate on a MALDI plate and analyzed on a 5800 MALDI-TOF-MS instrument (Applied Biosystems, Framingham, MA). Mass spectra of each spot were obtained by scanning from 800 to 4000 *m*/*z* in positive ion reflector mode. The instrument uses an Nd-YAG laser at 355 nm, and each spectrum is an average of 2400 laser shots.

## Results and discussion

### Evaluation of the effect of peptide length on toxin detection

The original peptide substrate used in the Endopep-MS method for BoNT/G detection was developed from the native sequence of VAMP-2 (L_60_–K_94_) that contained the cleavage site for BoNT/B (Q_76_F_77_) and BoNT/G (A_81_A_82_) [21]. The sequence of this peptide, VAMP-2_60–94_, was selected based on the investigation of the peptide as a BoNT/B substrate [32, 33], but the substrate had not been optimized for BoNT/G and there was insufficient research on its specificity and efficiency as a BoNT/G substrate and its application in an MS-based functional assay. To understand the VAMP-2 recognition and cleavage by BoNT/G, we initially examined the effect of peptide length of the substrate for the detection of BoNT/G cleavage products. Table 1 shows the relative production of cleavage products generated from BoNT/G cleavage of synthetic VAMP-2 peptide substrates as measured by MALDI-TOF mass spectrometry. When three additional residues (D_57_Q_58_K_59_) were added onto the N-terminus of the template, VAMP-2_60–94_, the amount of C-terminal(CT) product generated from the new substrate, VAMP-2_57-94_, reduced to 65% upon BoNT/G cleavage. Because both peptides share an identical sequence in their C-terminal region, a decrease in the measured CT product could be attributed to reduced substrate efficiency of the new peptide, explained through a reduction in substrate binding or enzyme cleavage or both of these mechanisms. Further extension by an additional 17 N-terminal residues in VAMP-2_41-94_ did not significantly alter the yield of the CT product. The result suggests that the tri-residue group of D_57_Q_58_K_59_ interferes with the interaction between BoNT/G and the substrate and that the N-terminal sequence beyond D_57_ may not participate in the substrate/toxin interactions. This is in contrast to the results observed in BoNT/B cleavage, where the addition of N-terminal residues 33–59 within VAMP-2 does not reduce the relative rates of cleavage by the BoNT/B enzyme [32].

**Table 1:** Relative production of various peptide substrates cleaved by BoNT/G toxin

Peptide	Sequence^a^	MH^+^ (Da)			Rel. product (%)^c^	
		Substrate	CT product	NT product	CT product	NT product
VAMP2_41–94_	EVVDIMRVNVDKVLERDQKLSELDDRADALQAGASQFETSAAKLKRKYWWKNLK	6304.3	1762.1	4561.2	51	
VAMP2_57–94_	DQKLSELDDRADALQAGASQFETSAAKLKRKYWWKNLK	4409.3	1762.1	2666.2	65	
VAMP2_60–94_	LSELDDRADALQAGASQFETSAAKLKRKYWWKNLK	4038.1	1762.1	2295.0	100	
VAMP2_63–94_	LDDRADALQAGASQFETSAAKLKRKYWWKNLK	3709.0	1762.1	1965.9	38	
VAMP2_66–94_	RADALQAGASQFETSAAKLKRKYWWKNLK	3365.8	1762.1	1622.7	56	
VAMP2_69–94_	ALQAGASQFETSAAKLKRKYWWKNLK	3023.7	1762.1	1280.6	24	
VAMP2_60–94_	LSELDDRADALQAGASQFETSAAKLKRKYWWKNLK	4038.1	1762.1	2295.0		100
VAMP2_60–91_	LSELDDRADALQAGASQFETSAAKLKRKYWWK	3682.9	1406.9	2295.0		51
VAMP2_60–87_	LSELDDRADALQAGASQFETSAAKLKRK	3019.6	743.6	2295.0		ND^b^

^a^Underlined letters represent the amino acid residues at the BoNT/G cleavage site

^b^The peak of the cleavage product was not detected

^c^The Rel. product (%) of each peptide was calculated as the percentage of the product area ratio (A_CT product_/A_IS_ or A_NT product_/A_IS_) of the peptide to the VAMP2_60–94 peptide_

In order to evaluate the effect of decreasing peptide length on substrate cleavage efficiency, three new peptides (VAMP-2_63–94_, VAMP-2_66–94_, VAMP-2_69–94_) with 3, 6, or 9 fewer residues than the VAMP-2_60–94_ peptide were tested as BoNT/G’s substrates. It was observed that these peptides resulted in less cleavage with approximately 50% or lower CT products than the VAMP-2_60–94_ substrate (Table 1), suggesting the need to conserve residue(s) in the L_60_S_61_E_62_ string to maintain high substrate efficiency. In comparison with BoNT/B cleavage, the VAMP-2_69–94_ resulted in a 24% rate of cleavage by BoNT/G while the BoNT/B cleavage efficiency of a similar peptide, VAMP-2_70–94_, reduced to approximate 4% [32], suggesting a different impact of the amino acids N-terminal to the cleavage site on the hydrolysis of the substrates by the two enzymes. This difference may be due to the fact that the BoNT/B cleavage site is five amino acids N-terminal from the BoNT/G cleavage site.

In addition to the N-terminal truncations, C-terminal truncations on VAMP-2_60–94_ were also evaluated. As shown in Table 1, the removal of N_92_L_93_K_94_ residues generated a peptide (VAMP-2_60–91_) that retained only half of the cleavage activity, implying the importance of the amino acids in this region of the VAMP-2 sequence. Further deletions in the string of residues Y_88_W_89_W_90_K_91_ (VAMP-2_60–87_) led to no detection of the NT product under the experimental conditions used. Considering that these residues are in close proximity to the BoNT/G cleavage site, A_81_A_82_, our data revealed that all or some combinations of these four C-terminal residues play a critical role in substrate binding and/or hydrolysis and their side chains might directly interact with the key groups in the catalytic cavity of BoNT/G enzyme. In contract, the absence of these residues in protein or peptide substrates resulted in no or moderate changes on the cleavage efficiency by full length or light chain of BoNT/B toxin [32, 34]. Again, this variation implies that type G and type B BoNTs possess different enzyme-substrate interactions, although their cleavage sites in the VAMP-2 substrate are only five amino acids apart. In conclusion, approximately 13 amino acid residues N-terminal to the scissile bond and 10 C-terminal residues are required for the efficient cleavage by BoNT/G toxin.

### Contribution of individual residues of the substrate to BoNT/G cleavage

**Fig. 1:**
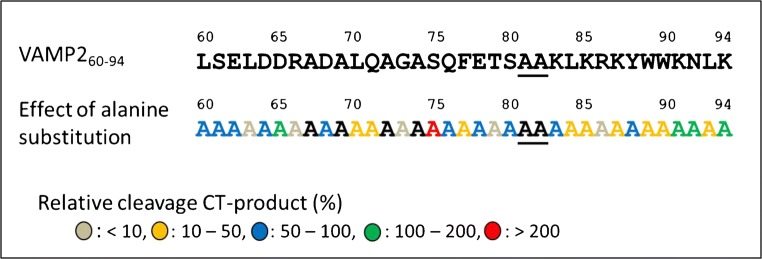
Cleavage efficiency of alanine-scanning peptides by BoNT/G. Each individual peptide was prepared by substituting a specific non-alanine residue on the Pep-1 with the alanine. The residues at BoNT/G cleavage site are underlined

VAMP-2_60–94_ produced the highest concentration of cleavage products among the peptides of various lengths tested, so we used this peptide as a template for further study and sequence optimization. In order to obtain general information about the contribution of individual residues to peptide function and their tolerance to mutation, an alanine-scanning method was used in which each non-alanine residue in Pep-1 (Fig. 1) was replaced with alanine. The substrate efficiency of these new peptides was analyzed by the Endopep-MS method. The effect of alanine substitution is depicted in Fig. 1 and is represented using the relative cleavage products of these peptides as generated by cleavage of the substrate by BoNT/G. For comparison of the Ala-substituted peptides, generation of the CT product rather than NT product was monitored, because a shorter CT product consisting of 13 residues usually yields a higher peak than its NT product counterpart (22 residues) in a MALDI-TOF mass spectrum.

Among 29 Ala-substituted residues, some substitutions inhibited or enhanced the substrate cleavage by BoNT/G, while others led to no or less change. Significant reduction (< 10%) of toxin cleavage was observed for five point mutations including L63A, R66A, G73A, T79A, and R86A, suggesting that these substitutions may cause a structural alteration that disrupts some of the substrate-enzyme interactions. On the other hand, substitutions of D65A, S75A, K91A, N92A, and K94A resulted in elevated substrate cleavage. In particular, the S75A mutant showed more than twofold increase in cleavage product implying the replacement of the polar serine residue to a non-polar alanine alters the local microenvironment and favors the peptide recognition and/or enzyme hydrolysis. The positive or negative effects of L63A, D65A, and R66A are consistent with the results described in the truncation study, that is, some of the N-terminal residues at the distal end of the scissile bond play important roles in substrate recognition. In addition, all of the substitutions of the C-terminal residues 83–90 gave rise to peptides with a lower detection of CT products. The susceptibility of these residues to changes may be due to their proximity to the C-terminus of the cleavage site. This is also in agreement with the result that the VAMP-2_60–87_ substrate, where C-terminal truncation greatly diminished the amount of BoNT/G, mediated cleavage (Table 1). It should be noted that because the cleavage efficiency of substituted peptides was determined by measuring the intensity of the CT product, the effect of C-terminal residue substitution or deletion could also be attributed to changed ionization efficiency of the corresponding CT products.

#### Optimization by internal substitutions and terminal modifications

In order to improve the sensitivity of the Endopep-MS method for the detection of active BoNT/G, we began to optimize the VAMP-2_60–94_ (renamed to Pep-1 thereafter) by sequentially substituting its residues with amino acids that have similar or different side-chain properties. The choice of substituents for each residue in Pep-1 was initially based on the Ala-scanning results and started from the C-terminal residues. Once the best substitution or deletion was determined, the modification was permanently incorporated into the location and the process was shifted to the left of the selected location. Table 2 lists some selected peptides that show higher detection of the cleavage CT product by MALDI-TOF MS due to sequence optimization using this method.

**Table 2:** Cleavage of selected peptides with multiple mutations by BoNT/G

Peptide	Sequence^a^								Area ratio^b^	Relative CT product
	60	65	70	75	80	85	90	94		
Pep-1	LSELDDRADALQAGASQFETSAAKLKRKYWWKNLK								0.18 ± 0.01	1.0
Pep-2	LSELDDRADALQAGASQFETSAAKLKRKYWWAKLK								0.57 ± 0.04	3.2
Pep-3	LSELDDRADALQAGASQFETSAAKLKRKYWWARLK								0.58 ± 0.05	3.2
Pep-4	LSELDDRADALQAGASQFETSAAKLKRKYWWAKL								0.80 ± 0.20	4.5
Pep-5	LSELDDRADALQAGASQFETSAAKLKRRYWWAKL								0.96 ± 0.02	5.3
Pep-6	LSELDDRADALQAGASQFESSAAKLKRRYWWAKL								0.95 ± 0.03	5.3
Pep-7	LSELDDRADALQAGATQFESSAAKLKRRYWWAKL								2.11 ± 0.08	11.7
Pep-8	LSELDDRADALQAGAAQFESSAAKLKRRYWWAKL								2.38 ± 0.39	13.2
Pep-9	LSELDDRADALQAGAKQFESSAAKLKRRYWWAKL								2.77 ± 0.03	15.4
Pep-10	LSELDDRADALQKGAKQFESSAAKLKRRYWWAKL								2.84 ± 0.12	15.8
Pep-11	LSELDDRADAL KGAKQFESSAAKLKRRYWWAKL								2.80 ± 0.08	15.6
Pep-12	LSELDDRADSL KGAKQFESSAAKLKRRYWWAKL								3.38 ± 0.25	18.8
Pep-13	LSELDDRAESL KGAKQFESSAAKLKRRYWWAKL								7.39 ± 0.70	41.1
Pep-14	LSELEERAESL KGAKQFESSAAKLKRRYWWAKL								8.56 ± 0.28	47.6
Pep-15	LDELEERAESL KGAKQFESSAAKLKRRYWWAKL								9.83 ± 0.14	54.7
Pep-16	KDELEERAESL KGAKQFESSAAKLKRRYWWAKL								10.51 ± 0.31	58.5

^a^Red letters represent substituted amino acid residues. Underlined letters are residues consisting of the cleavage site of BoNT/G. Gaps in some sequences indicate the deletion of the residue Q_24_

^b^Area ratios represent the ratio of the peak area of C-terminal products versus those of the internal standard peak. Data were obtained from experiments in triplicate

While no significant improvement was achieved by substituting the first two C-terminal residues with other amino acids, the K91A/N92K double mutant (Pep-2) performed 3 times better than the template Pep-1. Similar improvement was achieved by replacing N_92_ with another positive charged residue, Arg, in Pep-3. The lysine residue, however, was incorporated at this position for further optimization, because it appeared to facilitate the efficient synthesis of peptides and/or enhance the solubility of the peptide in the reaction solution. The same consideration applied to the substitutions of T79S (Pep-6), A72K (Pep-10), and L60K (Pep-16) and the deletion of Q_71_ (Pep-11). In addition, the removal of the K_94_ residue increased the intensity of the detected CT product from this peptide (Pep-4). This is consistent with the alanine-scanning result described above that the K94A substitution increased the cleavage efficiency by BoNT/G. In the native VAMP-2 protein, this residue is immediately adjacent to its transmembrane domain (VAMP-2_95–114_) and is unlikely that an enzyme such as BoNT/G can access the residue. In short peptides, however, this residue may become flexible and form new interactions with the enzyme to affect its binding affinity.

A conservative mutation of K87R in Pep-5 slightly increased the detection of the cleavage products and was selected. Replacement of polar serine (S_75_) with a non-polar alanine residue in Pep-8 resulted in a twofold increase in the detection of the C-terminal product, consistent with the results obtained from alanine-scanning study described above. Surprisingly, a similar degree of improvement was also obtained for the conservative mutation of S75T (Pep-7) and the substitution of S75K (Pep-9). These observations imply that the original serine at this position may be in contact with the enzyme in a way that does not favor the hydrolysis reaction and that this interaction is removed by any other substitution to increase the reaction activity.

Although most of the non-polar residues were irreplaceable, the substitution of alanine at 69 with polar serine (Pep-12) yielded about a 20% improvement. Substrate performance was substantially increased by a conservative replacement of the aspartic acid residue at the position of 68 with glutamic acid (Pep-13), implying an enzyme-substrate interaction network was enhanced by elongating the length of the carboxylic side chain. In addition, another two conservative mutants of D64E and D65E in Pep-14 also produced a better substrate than its precedent. Moreover, additional improvement was obtained with the replacement of S_61_ with an aspartic acid residue (Pep-15), suggesting that a negatively charged group is favored at this location.

General methods for optimizing peptide function include the following: (a) identifying special amino acid residues that significantly affect function by point mutation, alanine scanning, or structural analysis if the crystal/solution structure of an enzyme/substrate is available and (b) improving function by replacing these residues individually or together with different natural or unnatural amino acid residues. One of the limitations of these methods is that they do not fully consider the flexibility of peptide conformation at the time of substitution. Because of the short length of a peptide, any substituent may alter its overall conformation and thus change the contact of the peptide and the enzyme. Therefore, some residues that are originally identified as unimportant may become significant after replacing other residues and may need to be optimized, while the critical residues determined originally may change to less important ones. In this report, the BoNT/G substrate was optimized by one-by-one substitution walking throughout the entire sequence of the template. In comparison with traditional methods, this approach guarantees a higher degree of optimization, because more residues were taken into account and the structural changes caused by the substitution of some special residues were balanced by more alternatives. For example, the residue of Q_71_ in Pep-1 was determined by Ala-scanning to be important, but no functional loss was observed when the residue was deleted from Pep-10, which contains several substitutions (Table 2). On the other hand, alanine scanning revealed that the aspartic acid at position 68 was not an essential residue in Pep-1; however, a conservative replacement of this residue in the partially optimized peptide resulted in a twofold improvement. In summary, the application of this internal modification approach dramatically enhanced the detection of the toxin cleavage products by approximately 60-fold.

**Fig. 2:**
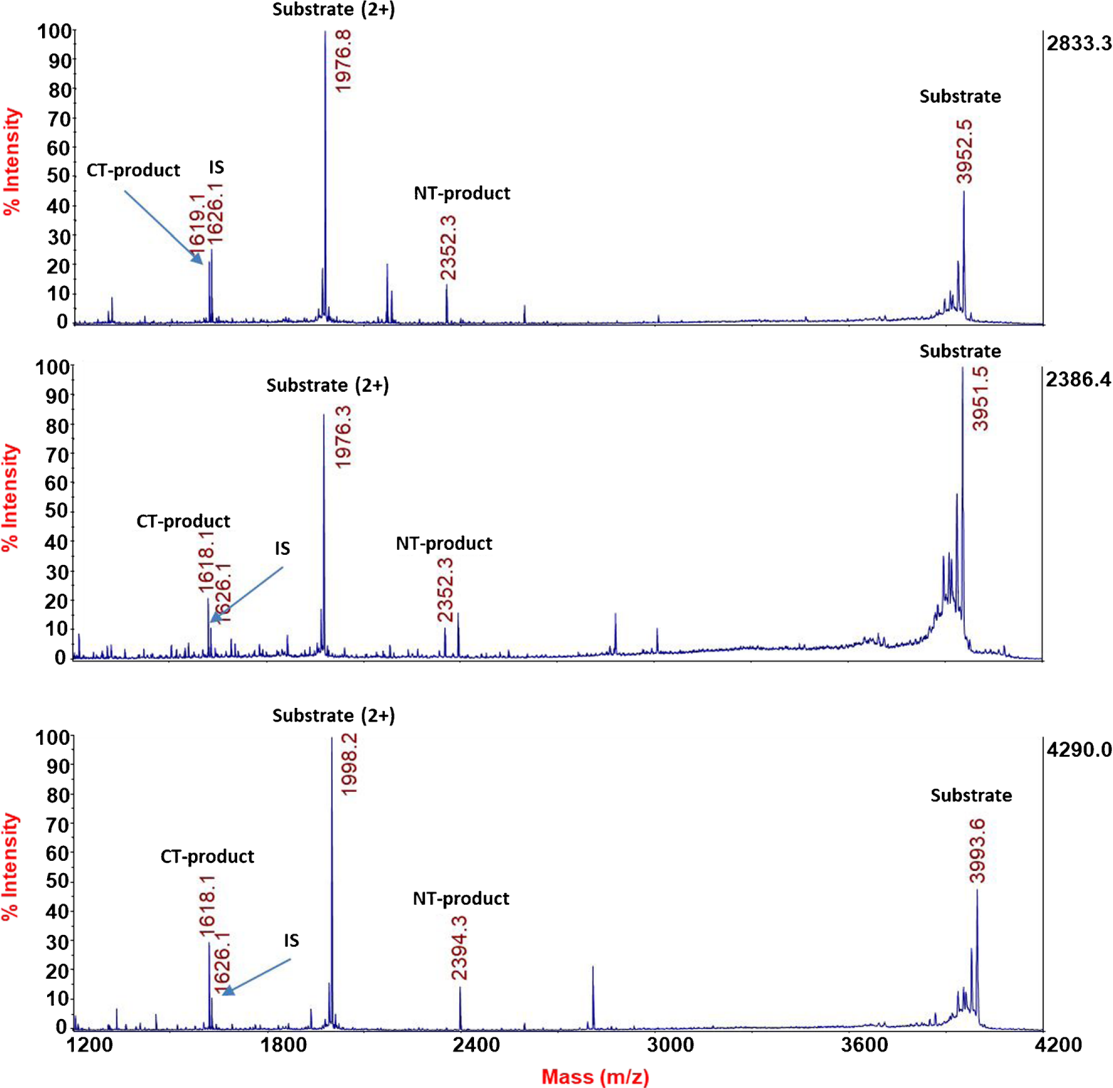
Mass spectra of the BoNT/G hydrolysis of the peptide substrates with various termini including Pep-16 without terminal protection (top), C-terminal-amidated Pep-17 (middle), and N-terminal-acetylated/C-terminal-amidated Pep-18 (bottom). IS, internal standard of the CT product

The terminal protection of a peptide with N-terminal acetylation and C-terminal amidation is two common modifications. These modifications can improve the stability of a peptide in biological matrices by blocking the unnatural charges at the end of the peptide and mimicking the amide bonds in native proteins. Figure 2 shows the mass spectra of two new peptides with either or both ends of Pep-16 protected. For C-terminal-amidated Pep-17, the intensity of the NT product peak relative to that of the unreacted substrate (2+) peak reduced slightly presumably due to decreased cleavage efficiency. However, the relative intensity of the CT product versus internal standard increased significantly, representing an elevated ionization of this fragment after replacing the C-terminal carboxyl group with a positively charged amino group. When the two terminal modifications were included (Pep-18), additional improvements in the peaks of the CT and NT products were observed, implying that the N-terminal acetylation enhanced the hydrolysis of the peptide.

**Fig. 3:**
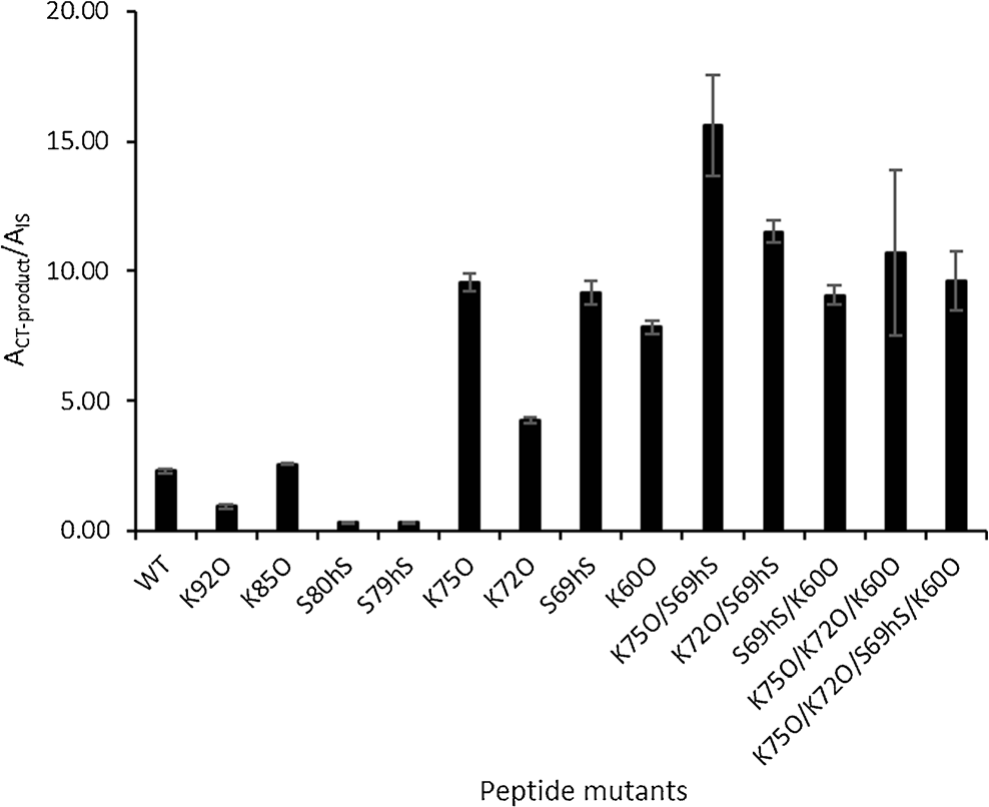
Cleavage of peptide mutants by BoNT/G. The mutants were constructed by substituting selected lysine (K) residue(s) with ornithine (O) and/or serine (S) residue(s) with homoserine (hS), respectively, on Pep-18 (WT), Ac-K^60^D_61_E_62_L_63_E_64_E_65_R_66_A_67_E_68_S_69_L_70_K_72_G_73_A_74_K_75_Q_76_F_77_E_78_S_79_S_80_A_81_A_82_K_83_L_84_K_85_R_86_R_87_Y_88_W_89_W_90_A_91_K_92_L_93_-NH_2_

Unnatural amino acids are often introduced into peptides to enhance their performance as inhibitors or substrates [24, 35]. In order to further improve the sensitivity of the Endopep-MS assay for BoNT/G activity detection, we constructed more peptide substituents with some unnatural amino acid residues based on the sequence of Pep-18. Two such amino acids, i.e., ornithine to replace lysine and homoserine to replace serine, were used because these substitutions were not expected to cause significant changes in peptide solubility and might not add additional challenges in peptide synthesis. As shown in Fig. 3, two single mutants, i.e., S79hS and S80hS where serine residues were substituted with homoserine, resulted in a dramatic reduction in the hydrolysis of the substrates. The main reason might be due to the location of these two residues being too close to the substrate scissile bond, A_81_A_82_, and therefore, a slight change could interrupt enzyme-substrate interactions. In addition, reduced cleavage occurred on another single mutation (K92O), where the second C-terminal lysine residue was replaced by ornithine.

On the other hand, several single mutants including K75O, K72O, S69hS, and K60O increased cleavage efficiency by approximately two- to fourfold, underlining the importance of further optimization with unnatural amino acids. Along with the described significant elevation in the detection of cleavage product with the mutants of S75A in VAMP-2_60–94_ in the alanine-scanning method, and S75T (Pep-7), S75A (Pep-8), and S75K (Pep-9) in mutation analysis, the substitution of the lysine residue at the same position by ornithine (K75O) further increased the cleavage of the peptide. This provides additional evidence to support the hypothesis that the side-chain group at this position may be located in the catalytic site of BoNT/G and that it is in direct contact or close to one or more enzyme residues.

Different effects were observed when two or more of these four mutations were combined. Double mutations of K75O/S69hS and K72O/S69hS elevated the cleavage of formed substrates, while the combination of S69hS and K60O did not yield any significant changes. Meanwhile, the addition of two single substitutions K60O and K72 in the K75O/S69hS double mutant caused adverse effects, and the gain obtained by the latter K75O/S69hS mutation was canceled. Given the fact that the peptide of K75O/S69hS produced the best results, this peptide (termed as Pep-19) was chosen as the optimal substrate for BoNT/G for the Endopep-MS activity assay.

#### Comparison of the newly optimized substrate with the original peptide

**Fig. 4:**
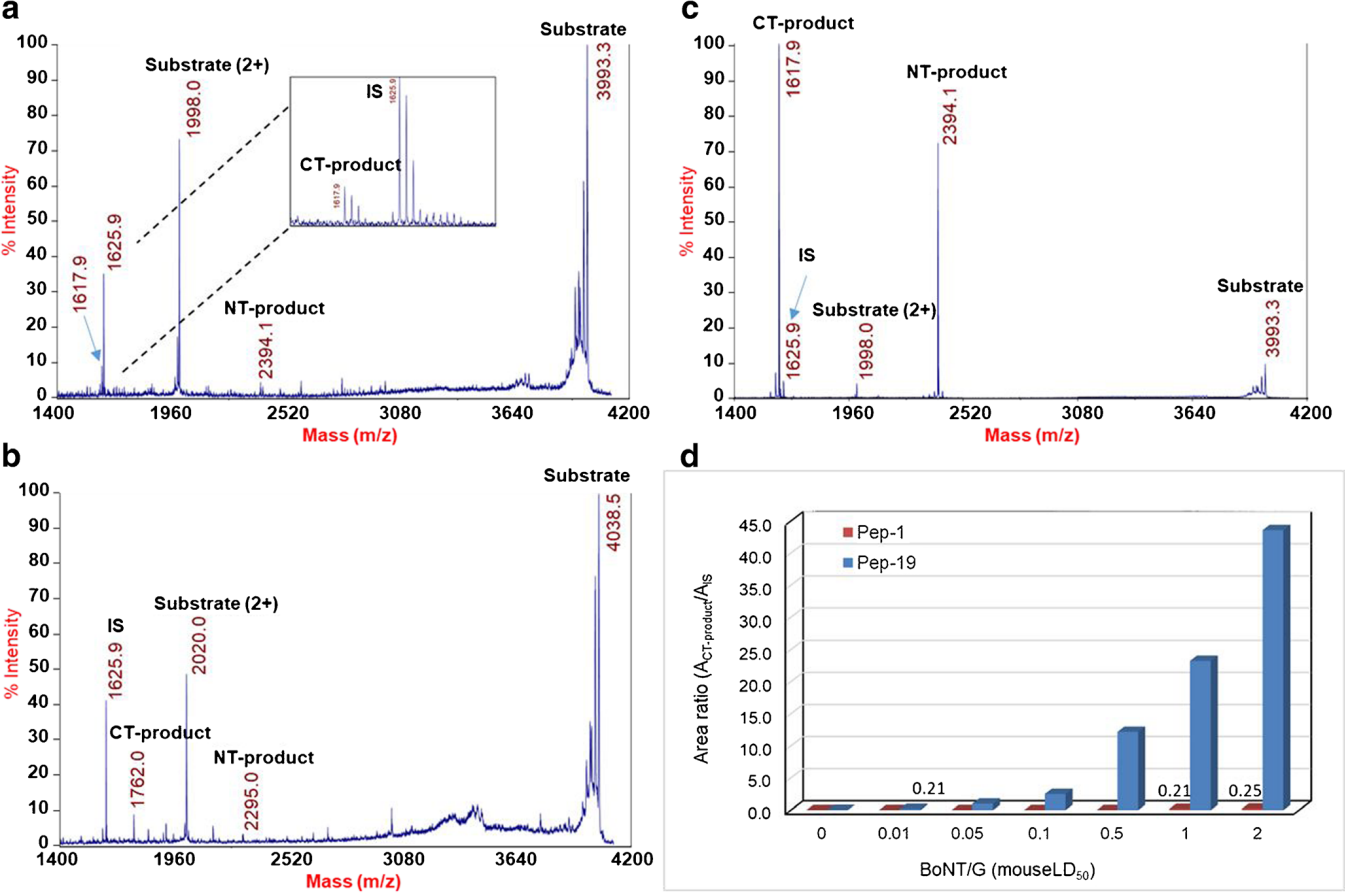
Comparison of the substrate efficiency of optimized peptide (Pep-19) and the old peptide (Pep-1). Mass spectra of the reaction solution with 0.01 mouseLD_50_ BoNT/G and Pep-19 (**a**), 2 mouseLD_50_ toxin and Pep-1 (**b**), and 2 mouseLD_50_ toxin and Pep-19 (**c**). Cleavage of the Pep-19 (blue) and Pep-1 (red) by BoNT/G toxin of various concentrations spiked in the reaction solution (**d**). The specific activity of non-activated BoNT/G complex was provided by the manufacturer. Cleavage reactions were conducted at 42 °C for 4 h

To assess the optimization effect, the optimized peptide (Pep-19) was compared with the original substrate (Pep-1) currently used in the Endopep-MS assay. As depicted in Fig. 4, the relative peak intensities of the products yielded from the cleavage of Pep-19 by 0.01 mouseLD_50_BoNT/G were similar to those obtained from the reaction of Pep-1 by the toxin with 2 mouseLD_50_, a 200-fold difference in toxin concentration (Fig. 4a, b). This was also demonstrated by the very high relative intensities of both CT and NT products in the spectrum of the cleavage of Pep-19 by 2 mouseLD_50_ toxin (Fig. 4c). These results indicate that the assay sensitivity was significantly improved with the optimized substrate. Quantitative analysis using various concentrations of the toxin resulted in the same conclusion (Fig. 4d). To further demonstrate the improved sensitivity of the Endopep-MS assay using the new peptide substrate, we compared the detection of the toxin at various concentrations (0–2 mouseLD_50_) with the two peptide substrates. As shown in Fig. 4d, the peak area ratio of the CT product and its internal standard is much higher with that of the optimized peptide substrate, indicating an improved ability of the toxin to cleave the new peptide substrate. In addition, specificity studies were conducted to examine whether other serotypes of BoNT can hydrolyze this peptide. It was observed that no cleavage products were detected by the mass spectrometry analysis of the cleavage reactions when Pep-19 was incubated with other serotypes of botulinum neurotoxins including BoNT/A, BoNT/B, BoNT/C, BoNT/D, BoNT/E, or BoNT/F at high concentrations (data not shown), indicating that this peptide was a substrate specific to BoNT/G but not to any other BoNT serotypes.

## Conclusion

Peptide substrates used in the Endopep-MS assay for the detection of BoNT/G were optimized through single and multiple substitution(s) and terminal modifications using the current Endopep-MS peptide as a template. The preference of the amino acid residues in the template was evaluated by alanine scanning, and this provided useful information for the optimization of the peptide substrate. The formation of cleavage products was significantly improved upon the substitution or deletion of several internal residues. Introduction of some unnatural amino acids in the modified peptides further enhanced the hydrolysis of new substrates by BoNT/G and/or the detection of cleavage products. In comparison with the original substrate, the final best peptide showed an increase in sensitivity compared with the legacy BoNT/G assay by two orders of magnitude, resulting in a toxin detection level of as low as 0.01 mouseLD_50_.
